# A Case Report of Delayed Opioid Toxidrome After Administration of Naloxone

**DOI:** 10.5811/cpcem.6622

**Published:** 2024-05-29

**Authors:** Maiya Cowan, Prasanna Kumar, Jenny McManus, Sean Bilodeau, Andrew Beck

**Affiliations:** * Brown University, Providence, Rhode Island; † Lifespan/Rhode Island Hospital, Department of Emergency Medicine, Providence, Rhode Island

**Keywords:** *naloxone*, *opioid*, *overdose*, *fentanyl*, *case report*

## Abstract

**Introduction:**

Opioid use is an epidemic that plagues the United States. Patients frequently present to the emergency department (ED) after opioid toxicity, which can lead to respiratory failure, apnea, and death. Although there is an effective antidote, naloxone, the current guidelines surrounding post-naloxone administration monitoring are loosely defined.

**Case Report:**

We present a case in which an individual was administered naloxone after an intentional opioid overdose and was monitored for four hours, as is standard in our institution. He remained in the ED for additional workup following this observation period and subsequently experienced signs of severe respiratory depression, requiring bag-valve-mask ventilation, naloxone, and admission. Had he been discharged, as is typical after a four-hour observation period, the consequences could have been fatal. We present multiple theories as to why his opioid toxidrome may have presented in a delayed manner, including ingestion of fentanyl analogues and variability in metabolization of both opioids and naloxone. We also explore alternative overdose antidote products approved by the US Food and Drug Administration, which may impact post overdose care.

**Conclusion:**

This case suggests that the correct amount of time to monitor patients after naloxone administration may be longer than originally thought. Our aim in this article was to further the discussion regarding the most appropriate observation period in cases of opioid toxicity.

Population Health Research Capsule What do we already know about this clinical entity?

*The opioid epidemic is a significant cause of morbidity and mortality in the United States. Naloxone is a readily available antidote that is clinically effective.*

What makes this presentation of disease reportable?

*The patient suffered from opioid toxicity well after the established observation period for naloxone use at our institution ended.*

What is the major learning point?

*There may not be a universal observation period for opioid toxicity in the emergency department, as individuals may metabolize naloxone and opioids differently.*

How might this improve emergency medicine practice?

*This case may encourage clinicians to consider a longer observation period or maintain a higher threshold for discharging a patient after an opioid overdose.*

## INTRODUCTION

The opioid epidemic remains a serious public health crisis in the United States (US). Opioid overdoses account for a large proportion of emergency department (ED) visits nationwide and are the number one cause of death in individuals between 25–64 years of age.^1^ ^,^ ^2^ In 2021, 80,411 deaths involving opioid overdose were reported in the US, an increase from 68,630 the year prior.^3^ Naloxone is a competitive mu opioid receptor antagonist used to reverse the central and peripheral effects of opioid toxicity.^2^ Currently, there is no consensus regarding the duration of observation after naloxone administration. Various studies have cited between 1–4 hours as sufficient to prevent adverse events such as recurrence of respiratory depression, providing the patient has normal vital signs and mentation.^4^ ^,^ ^5^ However, some authors recommend even longer observation periods.^6^ This report highlights a case of delayed opioid toxidrome after naloxone administration despite an observation period of four hours, shedding light on potential pitfalls with current practice.

## CASE REPORT

A 30-year-old male with past medical history of polysubstance use disorder and asthma presented to the ED following an intentional intranasal opioid overdose with illicitly purchased fentanyl. He was found unresponsive, and an ambulance was called. En route, he received a total of six milligrams (mg) of intranasal (IN) naloxone and 0.4 mg intravenous (IV) naloxone to reverse the apneic state. This was his first time using fentanyl, and he denied any known co-ingestion. He did not take any daily medications or have any allergies.

Initial vital signs were as follows: heart rate 116 beats per minute; blood pressure 184/118 millimeters of mercury; temperature 97° Fahrenheit, and oxygen saturation of 94–100% on two liters of oxygen. On exam, the patient was mildly somnolent but arousable, nodding off during the interview with a Glasgow Coma Scale (GCS) of 15. His pupils were pinpoint. Thought content included active suicidal ideation (SI) with a plan. He demonstrated no signs of trauma. Urine drug screen, an immunoassay, was positive only for fentanyl, cannabinoids, and cocaine. This was later confirmed with an extended drug panel, which was also negative for the following: methadone, benzodiazepines, buprenorphine, amphetamines, oxycodone, opiates, and phencyclidine. Acetaminophen, ethanol, toxic alcohol, and salicylate levels were not detected. No other lab abnormalities were present.

The patient was given one liter of normal saline and placed on telemetry with constant observation. After four hours, the patient was medically stable as he was awake, alert, and cooperative with a GCS of 15. At this point, he demonstrated no signs of respiratory depression, pupillary abnormality, or mental status change. He remained in the ED for psychiatric evaluation in the setting of active SI. During this time, he was under constant in-person observation and handcuffed to his stretcher (arrested on scene). Approximately 5.5 hours after initial naloxone administration, the patient became apneic and unresponsive. He was ventilated via bag-valve-mask and given two mg of IN naloxone with prompt clinical response. He was started on an IV naloxone infusion at 0.4 mg/hour and admitted to the intensive care unit.

He was on the infusion for approximately two hours when it was discontinued. Approximately three hours after cessation of the infusion, he had another episode of apnea. An IV naloxone infusion was reinitiated with a 0.2 mg IV bolus, again with appropriate clinical response. He was successfully weaned off IV naloxone later that night. He was discharged to a correctional facility with psychiatric follow-up two days later.

## DISCUSSION

Deaths from opioid overdose have more than tripled over the last decade.^3^ As ED visits for opioid-related overdoses continue to rise, it is paramount to have standardized treatment protocols in place. Intranasal naloxone has gained momentum in the prehospital and community settings, even becoming available over the counter in 2023. Despite its widespread use, there continues to be disagreement over the most appropriate observation period to prevent adverse events.

The elimination half-life of IV naloxone ranges from 20–90 minutes, whereas that of many opioids can be substantially longer.^5^ For example, pharmaceutical fentanyl has a half-life of approximately 3.6 hours; and although human studies are lacking, some animal studies suggest that carfentanil can have a half-life of up to 7.7 hours.^7^ ^,^ ^8^ ^,^ ^9^ As a result, various time periods have been suggested as the most appropriate for observation after treatment with naloxone to prevent recurrence of toxicity. There are studies that cite one hour as safe and others that recommend observation periods of up to four hours.^4^ ^,^ ^5^ ^,^ ^10^ The patient was observed for four hours in keeping with institutional policy and subsequently recrudesced, which could have proven fatal.

There are many hypotheses as to why this patient presented atypically after opioid intoxication: variability in naloxone metabolism, ingestion of fentanyl analogues, and variability in opioid metabolism. The elimination of naloxone follows first order kinetics.^11^ Consequently, after four to five half-lives, 93.75–97% of the drug is eliminated, allowing for negligible remaining effect. Based on the well documented half-life range of 20–90 minutes, the rate of elimination among two people on either end of the spectrum can be approximately 4.5 times faster or slower.^5^ This is a substantial difference. Based on this range, if our patient were a fast metabolizer of naloxone, he likely would have required repeat doses within the observation period. However, if he were a slow metabolizer, he may have had enough naloxone to pass a four-hour observation period but would experience rebound toxicity once it wore off. There is scarce data reviewing this specific topic.

One recent study suggests a prolonged observation period between 6–12 hours in chronic opioid users or those who have ingested methadone, buprenorphine, or other long-acting opioids. Additionally, once 5 mg or more of IV naloxone is administered, admission is recommended.^6^ The rationale is that this increased dose may reflect the potency and half-life of the opioid ingested. Our case may be another data point to support the notion that there may be a role for dose-dependent observation periods after naloxone administration. This may be hard to quantify as dosing is based on clinical response and may overestimate the minimum amount of naloxone required for adequate reversal. In addition, this would require a method of standardization when current observation periods already vary greatly from facility to facility.^5^ ^,^ ^10^

Another explanation is that the patient unintentionally ingested a long-acting opioid, such as a fentanyl analogue. Fentanyl analogues and novel synthetic opioids have infiltrated the unregulated illicit drug market.^7^ Data regarding pharmacokinetic and pharmacodynamic properties of fentanyl analogues/novel synthetic opioids is scarce, and much of the data come from animal studies. One example, carfentanil, has permeated throughout the illicit drug market and may be associated with increasing overdoses in the US.^7^ One small cohort study of human subjects that analyzed postoperative pain found that fentanyl is 50–100 times more potent than morphine, while carfentanil is 10,000 times more potent than morphine.^7^ This is significant because potency, affinity for mu opioid receptors, and ease of dissociation can influence how much naloxone is needed to reverse opioid toxicity.^8^ Most relevant to this case, the half-lives of these compounds vary greatly and would have significant implications for the duration of monitoring.

The half-life of pharmaceutical fentanyl is 219 minutes (∼3.6 hours), and animal data suggests that the half-life of carfentanil is 7.7 hours.^7^ ^,^ ^9^ ^,^ ^12^ Given that the half-life of naloxone is 20–90 minutes, these compounds can be present for much longer than the reversal agent, resulting in recurrence of toxicity and need for longer monitoring times. To illustrate, one case series highlights 18 patients who experienced exaggerated opioid toxicity after testing positive for fentanyl on a limited drug screen. Seventeen of these patients required naloxone boluses, with four requiring prolonged infusions (26–39 hours). Furthermore, one patient experienced recurrent toxicity eight hours after naloxone discontinutation,^10^ similar to the patient presented in this case report. This sheds light on the need for more data regarding fentanyl and its analogues and potentially increasing the time for observation after fentanyl ingestions specifically.

Consideration was also given to the possibility that he had self-administered fentanyl in the ED. However, this was unlikely as he always had a staff member present due to SI and was in handcuffs. Another explanation for delayed opioid toxidrome could be variable metabolization of the drug. Fentanyl is primarily metabolized by cytochrome P450 3A4 (CYP450 3A4) in the liver and, to a lesser extent, duodenal microsomes and renally.^13^ Fentanyl is eliminated through conversion to inactive, nontoxic metabolites.^7^ As with any enzyme, metabolic function may be subject to variability among individuals with respect to expression and drug interactions. For example, patients with hepatic impairment will likely have decreased clearance of these medications. Additionally, drugs that compete with fentanyl can result in unforeseen interactions. To illustrate, benzodiazepines are also metabolized by CYP450 3A4, and co-ingestion could result in delayed conversion of fentanyl into inactive metabolites.^13^ As a result, if metabolism of the opioid was slower than that of naloxone then this patient could have experienced rebound toxicity once naloxone effects had diminished or waned. However, this patient specifically did not have any laboratory evidence of hepatic impairment or benzodiazepine co-ingestion.

While naloxone is the standard of care for opioid overdose reversal, other emerging products in the pharmaceutical sector may address breakthrough presentations similar to this case. For instance, in 2023 the US Food and Drug Administration approved nalmefene as an intranasal alternative to naloxone. While nalmefene and naloxone are both opioid antagonists, the benefit of nalmefene is that its half-life is considerably longer than naloxone at 11.4 hours.^14^ More studies are needed to compare its efficacy and side-effect profile to naloxone. However, it may serve as a potential alternative for opioid overdoses.

## CONCLUSION

This discussion highlights a case of delayed opioid toxidrome more than four hours after naloxone administration. The mechanism of these findings is unclear but may involve ingestion of fentanyl analogues or inter-individual variability in metabolization of opioids or naloxone. While data is limited on this phenomenon, this case highlights the need for more controlled studies on appropriate duration for monitoring after naloxone administration.

The authors attest that their institution requires neither Institutional Review Board approval, nor patient consent for publication of this case report. Documentation on file.
